# Supplementary material for: Frequency of Convenience Cooking Product Use Is Associated with Cooking Confidence, Creativity, and Markers of Vegetable Intake
Source: Nutrients. 2023 Feb 15;15(4):966. doi: 10.3390/nu15040966 (PMC9967409; doi:10.3390/nu15040966)
Supplement: Supplementary file 1 [file nutrients-15-00966-s001.zip › nutrients-2196828-supplementary.pdf]

SUPPLEMENT

Supplementary Table S1 – demographics by use of meal and recipe bases.

	Meal and Recipe Bases				Simmer Sauces				Pasta Sauces			
	>Weekly	Weekly	<Weekly	X^2 (p)	>Weekly	Weekly	<Weekly	X^2 p	>Weekly	Weekly	<Weekly	X^2 p
**Sex**												
Male	112 (49.3)	232 (47.0)	136 (43.5)	14.8 (0.1)	101 (44.1)	255 (49.1)	124 (43.4)		99 (47.8)	245 (46.0)	136 (46.3)	
Female	112 (49.3)	260 (52.6)	174 (55.6)		124 (54.1)	262 (50.5)	160 (56.0)	16.9 (0.05)	105 (50.7)	284 (53.3)	157 (53.4)	14.4 (0.1)
Others	3	2 (0.0)	3 (0.0)		4 (1.6)	2 (0.2)	2 (0.6)		3 (0.9)	4 (0.6)	1 (0.3)	
**Income**												
<$35,000	42 (18.5)	72 (14.6)	74 (23.6)	44.1 (0.0006)	38 (16.6)	87 (16.7)	63 (22.0)		45 (21.7)	74 (13.9)	69 (23.5)	
$35,000-$49,999	28 (12.3)	84 (17.0)	42 (13.4)		30 (13.1)	78 (15.0)	46 (16.1)		23 (11.1)	89 (16.7)	42 (14.3)	
$50,000-$74,999	47 (20.7)	86 (17.4)	55 (17.6)		47 (20.5)	86 (16.6)	55 (19.2)		40 (19.3)	81 (15.2)	67 (22.8)	60.2 (<0.0001)
$75,000-$149,999	26 (11.5)	83 (16.8)	41 (13.1)		25 (10.9)	80 (15.4)	45 (15.7)	45.6 (0.0003)	30 (14.4)	84 (15.8)	36 (12.2)	
$150,000-$199,999	49 (21.6)	110 (22.3)	59 (18.9)		50 (21.8)	125 (24.1)	43 (15.0)		42 (20.3)	133 (25.0)	43 (14.6)	
>$200,000	22 (9.7)	20 (4.1)	13 (4.2)		22 (9.6)	27 (5.2)	6 (2.1)		19 (9.2)	27 (5.1)	9 (3.1)	
Declined to answer	13 (5.7)	39 (7.9)	29 (9.3)		17 (7.4)	36 (6.9)	28 (9.8)		8 (3.8)	45 (8.4)	28 (9.5)	
**Hours worked/week**												
<15	85 (37.4)	220 (44.5)	159 (50.8)	32.9 (0.0001)	84 (36.7)	222 (42.8)	158 (55.2)		70 (33.8)	229 (43.0)	165 (56.1)	
15-30	46 (20.2)	91 (18.4)	58 (18.5)		47 (20.5)	102 (19.7)	46 (16.1)	46.2 (<0.0001)	44 (21.3)	104 (19.5)	47 (16.0)	48.4 (<0.0001)
30-50	88 (38.8)	173 (35.0)	90 (28.7)		88 (38.4)	183 (35.3)	80 (28.0)		88 (42.5)	188 (35.3)	75 (25.5)	
50+	8 (3.5)	10 (2.0)	6 (1.9)		10 (4.3)	12 (2.3)	2 (0.6)		5 (2.4)	12 (2.3)	7 (2.4)	
**Education**												
<Year 12[#]	27 (11.9)	62 (12.6)	45 (23.6)	29.1 (0.02)	23 (10.0)	64 (12.3)	47 (16.4)		22 (10.6)	63 (11.8)	49 (16.7)	
Year 12[#]	41 (18.1)	104 (21.1)	62 (19.8)		49 (21.4)	103 (19.8)	55 (19.2)		50 (24.2)	106 (16.7)	51 (17.3)	
Technical diploma	61 (26.9)	148 (30.0)	91 (29.1)		59 (25.8)	156 (30.1)	85 (29.7)	26.8 (0.03)	44 (21.3)	166 (15.2)	90 (30.6)	31.2 (0.008)

Bachelor’s degree	51 (22.5)	127 (25.7)	77 (24.6)		54 (23.6)	137 (26.4)	64 (22.4)		53 (25.6)	130 (24.4)	72 (24.5)	
Postgrad degree	44 (19.4)	50 (10.1)	33 (10.5)		40 (17.5)	55 (10.6)	32 (11.2)		36 (17.4)	61 (11.4)	30 (10.2)	
Declined	3 (1.3)	3 (0.6)	5 (1.6)		4 (1.7)	4 (0.7)	3 (1.0)		2 (1.0)	7 (1.3)	2 (0.6)	
Nights/week cooking at home												
>7	90 (39.6)	171 (34.6)	123 (39.3)	24.2 (0.02)	95 (41.5)	177 (34.1)	112 (39.2)		82 (39.6)	185 (34.7)	117 (39.8)	
5-6	77 (33.9)	145 (29.4)	91 (29.1)		74 (32.3)	157 (30.2)	82 (28.7)		57 (27.5)	168 (31.5)	88 (29.9)	
3-4	50 (22.0)	127 (25.7)	70 (22.3)		45 (19.7)	135 (26.0)	67 (23.4)	22.8 (0.03)	50 (24.2)	134 (25.1)	63 (21.4)	19.3 (0.03)
1-2	9 (4.0)	48 (9.7)	28 (8.9)		13 (5.7)	48 (9.2)	24 (8.4)		17 (8.2)	42 (7.9)	26 (8.8)	
<1	1 (4.4)	3 (0.6)	1 (0.0)		2 (0.8)	2 (0.3)	1 (0.3)		1 (0.4)	4 (0.7)	0 (0.0)	
Ages												
18-24	39 (17.3)	80 (16.2)	44 (14.1)	134 (0.0001)	48 (21.0)	82 (15.8)	33 (11.6)		50 (24.2)	76 (14.2)	37 (12.7)	
25-34	62 (27.4)	83 (16.8)	45 (14.5)		55 (24.0)	89 (17.2)	46 (16.2)		50 (24.2)	102 (19.1)	38 (13.0)	
35-44	59 (26.0)	71 (14.4)	43 (13.8)		47 (20.5)	95 (18.3)	31 (10.9)	122.2 (<0.0001)	49 (23.7)	93 (17.5)	31 (10.6)	149.6 (<0.001)
45-54	25 (11.0)	78 (15,8)	57 (18.3)		28 (12.2)	85 (16.4)	47 (16.5)		24 (11.6)	80 (15.0)	56 (19.2)	
55-65	16 (7.0)	83 (16.8)	54 (17.4)		22 (9.6)	74 (14.3)	57 (20.1)		18 (8.7)	87 (16.3)	48 (16.4)	
65+	25 (11.1)	99 (20.0)	68 (21.9)		29 (12.7)	93 (18.0)	70 (24.6)		16 (7.7)	94 (17.7)	82 (28.1)	

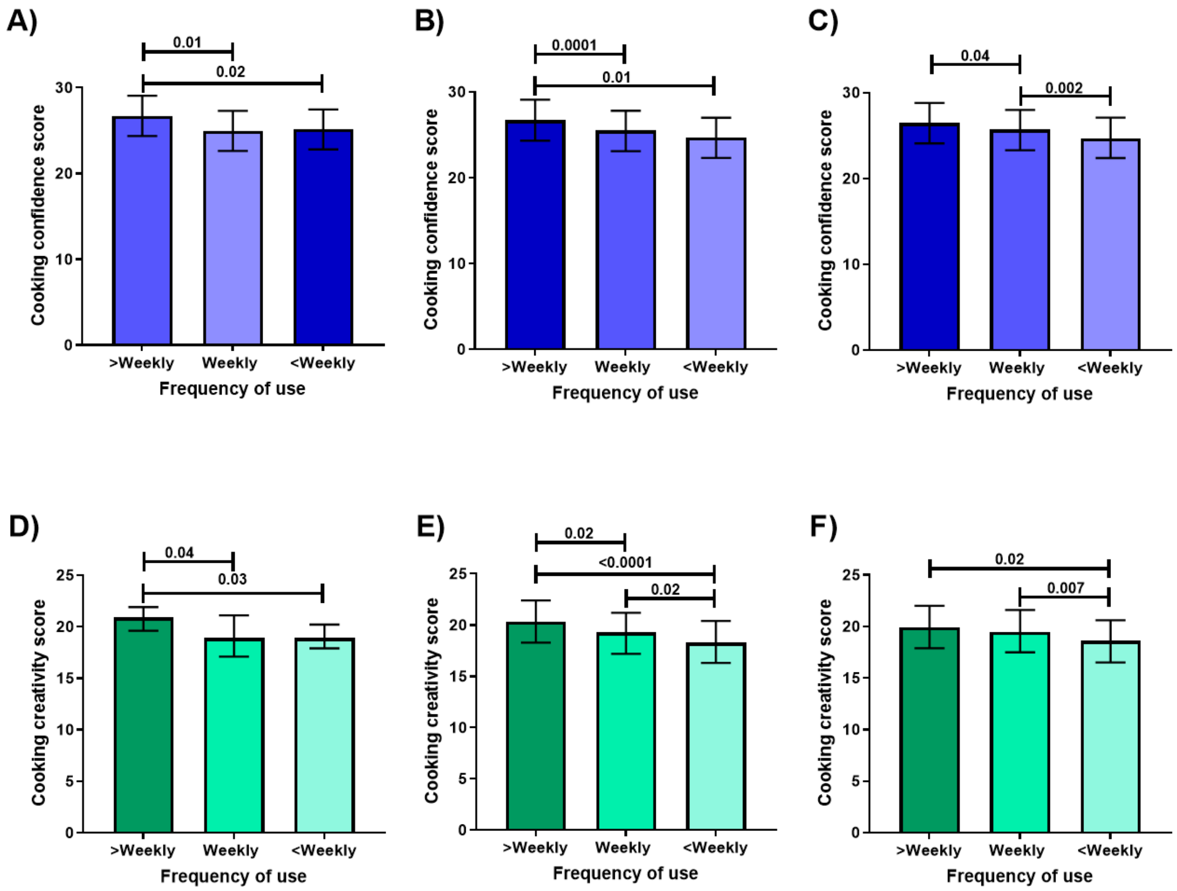

Supplementary Figure S1: ***Cooking confidence & creativity scores by frequency of convenience cooking product use (adjusted models);***
Cooking confidence scores by frequency of convenience cooking product use A) meal and recipe bases B) Simmer sauces C) Pasta sauces, adjusted for age, sex, income, education, work hours and frequency of cooking. Cooking creativity scores by frequency of convenience cooking product use D) meal and recipe bases E) Simmer sauces F) Pasta sauces adjusted for age, sex, income, education, work hours and frequency of cooking

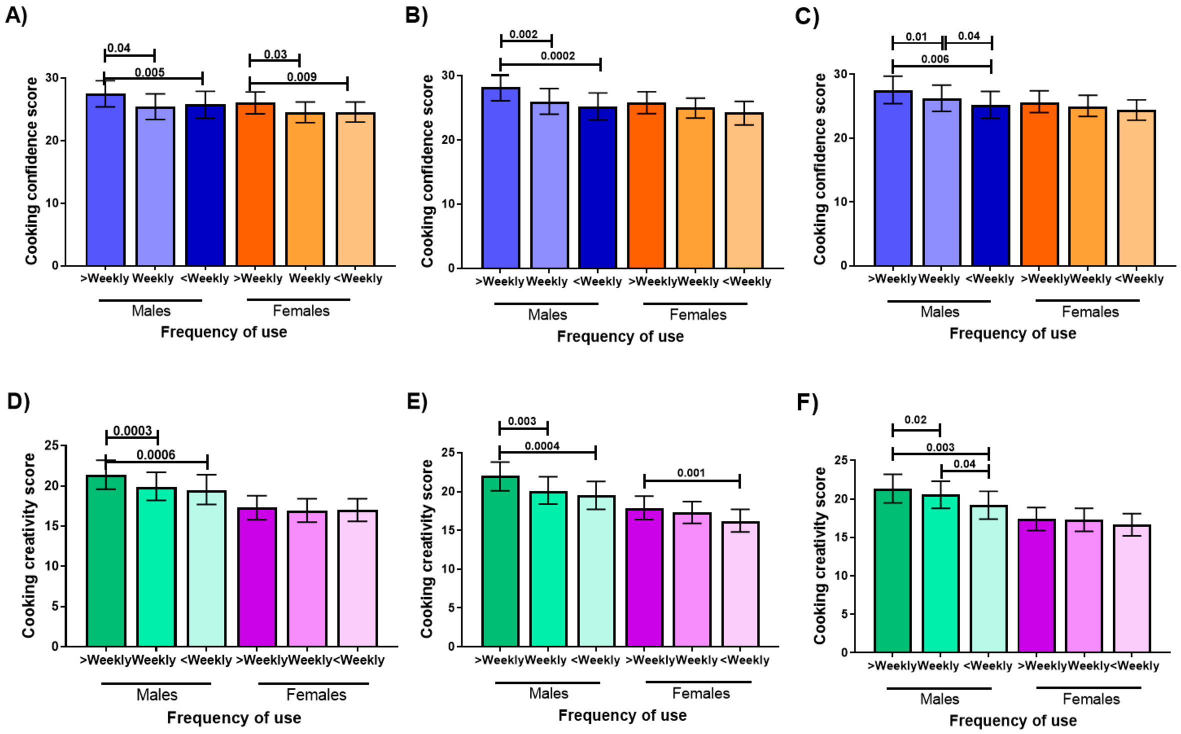

Figure S2: ***Cooking confidence & creativity scores by frequency of convenience cooking product use (sex- stratified models);*** *Cooking confidence scores by frequency of convenience cooking product use A) meal and recipe bases B) Simmer sauces C) Pasta sauces, stratified by sex and adjusted for age, sex, income, education, work hours and frequency of cooking. Cooking creativity scores by frequency of convenience cooking product use D) meal and recipe bases E) Simmer sauces F) Pasta sauces, stratified by sex and adjusted for age, sex, income, education, work hours and frequency of cooking.*

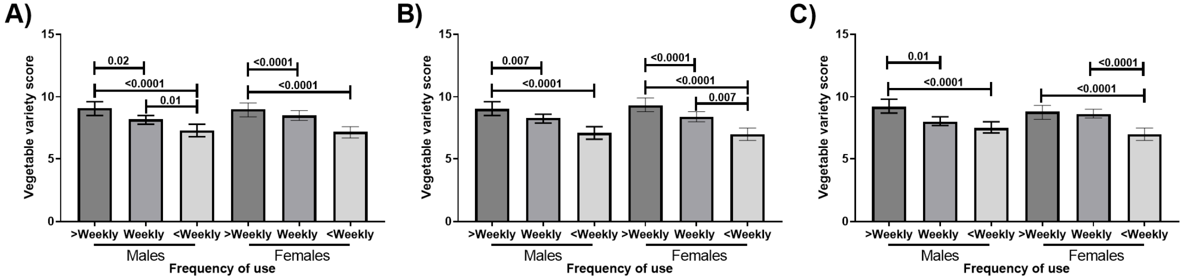

Supplementary Figure S3: ***Vegetable variety scores by frequency of convenience cooking product use (sex- stratified models);*** *Vegetable variety scores by frequency of convenience cooking product use A) meal and recipe bases B) Simmer sauces C) Pasta sauces, stratified by sex*

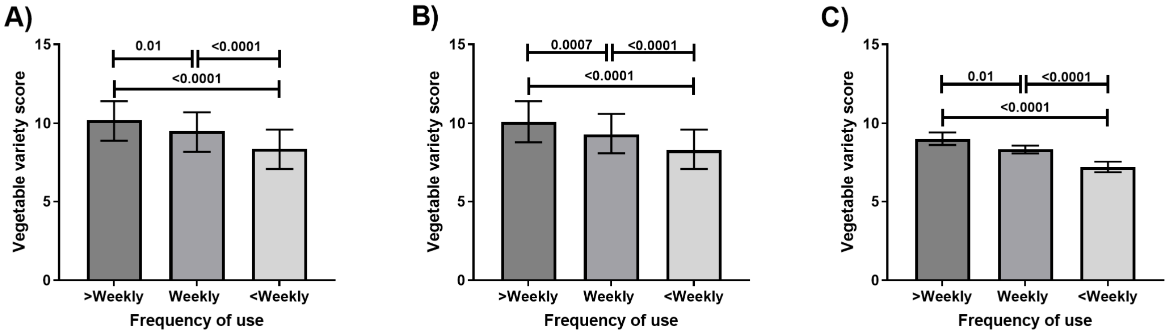

Supplementary figure S4: ***Vegetable variety scores by frequency of convenience cooking product use (adjusted models);*** *Vegetable variety scores by frequency of convenience cooking product use A) meal and recipe bases B) Simmer sauces C) Pasta sauces.*
